# A view forward from ten years of *BMC Biology*

**DOI:** 10.1186/1741-7007-11-32

**Published:** 2013-04-15

**Authors:** Miranda Robertson

## 

When *BMC Biology *was launched ten years ago, the open access movement in biomedical publishing was only just beginning to gain ground, the impact of Otto Warburg on cancer research had yet to be felt, parallels between human and fruit fly in the regulation of metabolism were just starting to accumulate, and the Hedgehog signaling pathway was not fully understood, still less exploited. In this article collection to mark the tenth anniversary of the publication of *BMC Biology*, we revisit all these topics and more, in a selection that reflects some of the more highly cited papers published by the journal over the decade of its existence.

In an age in which things change so fast that the future seems constantly in our face before we have finished with the present, it is a reasonable assumption that the appetite of readers for recollection will be limited. So none of the articles in our collection is just a reminiscence: all provide a selective overview of what has happened since, and an idea of what may come; and we will commit only the briefest word or two to the history of *BMC Biology*, as follows.

*BMC Biology *grew organically out of its subject-specific siblings of the BioMed Central series of journals, drawing on their Editorial Boards for its Editorial Board, and selecting from the papers submitted to these journals those that reviewers considered of sufficient interest or importance to be worth drawing to the attention of a wider readership. Once papers started to be submitted directly to *BMC Biology* this quickly became reciprocal, and good papers that did not seem quite to meet our criteria of breadth of interest could be published with a minimum of delay in a sister journal. Three years ago, *BMC Biology *fused [1] with its non-series sister *Journal of Biology*, which brought with it a tradition of commissioned review and comment, and an experimental policy, re-review opt-out, whose origins and development are revisited below.

All of the articles here are written by *BMC Biology *authors, or Editorial Board members, or both; or by us.

## Pit bulls, pussycats, peer review and the origins and future of open access

The first five articles in our collection are all, to a greater or lesser extent, about peer review, which is a perennial concern of authors and a daily issue for editors. Patrick O Brown, whom we interviewed on the revolutionary beginnings of open access [2], never thought the present system of peer review worked, and still doesn't: his vision at the start of the open access movement, inspired by the need for intelligent high-tech exploitation of large datasets, remains unfulfilled. For now.

Re-review opt-out, which is our relatively conservative answer to some of the more serious problems arising in the existing peer review process, was initiated in response to a single incident, recapitulated here by Peter Walter [3], whose account of his infuriating experience with another (highly respected) journal ends with a calmer reflection on the proper roles of reviewer and editor. The present operation of our policy is explained in the editorial in Q&A format that we published in February and which is reproduced here [4].

The revolution that Pat Brown projects in peer review is not going to be with us tomorrow, and in the meantime we must make the existing system work, so it is encouraging that Virginia Walbot's protocol for training postdocs in judicious refereeing, published in 2009 [5], has been so enthusiastically received: this is also reproduced here, with an update [6] in which she apologizes to dog-lovers offended by the species she chose for her (extremely effective) metaphor, and offers an alternative.

## Cancer, metabolism, flies, humans and Hedgehog

The three longer articles published here were commissioned for the anniversary collection to reflect some of our most cited - and, inevitably, less recent - research papers: shorter updates on some well cited papers published over more recent years illustrate the diversity of our publications - and, as noted by Penelope Austin and Kester Jarvis in their separate introduction to these papers [7] - the impact of high-throughput technology.

Not surprisingly, the three older and most cited publications include the paper from the laboratories of Grahame Hardie and Dario Alessi reporting the connection between the tumor suppressor LKB1 and the energy sensor AMPK that we published ten years ago just at the start of the surge of interest in the metabolism of tumor cells. Nor is it surprising that the paper opened at least as many questions as it answered. Hardie and Alessi realized at the time that because AMPK restores energy homeostasis in ATP-depleted muscle (for example) it might be a target for anti-diabetic drugs, and quickly established that metformin, very widely used in the treatment of type 2 diabetes, does indeed activate AMPK. The next question, given that LKB1, the physiological activator of AMPK, is a tumor suppressor, was whether metformin inhibits tumorigenesis. Studies on type 2 diabetes patients suggest that it does. But as Hardie and Alessi explain in their review [8], the evidence that has accumulated since has thrown up paradoxes to be resolved, as well as an explosion of new avenues to be explored.

A second highly cited paper was one from Norbert Perrimon's lab on RNAi, but he was not inclined to write on that, and we settled instead for an article on a more recent preoccupation of his with the exploration of metabolism using *Drosophila *as a surprisingly human-homologous model organism - an area in which *BMC Biology *has published a number of fine papers. He and Akhila Rajan review the accumulating evidence of similarities betwen us and fruit flies, and some recent insights that flies have delivered [9].

The story of screening for small-molecule modulators of the Hedgehog signaling pathway, originating in a paper from Jeff Porter's laboratory published a little over ten years ago [10], is told by Tom Carney and Philip Ingham, who cannot resist pointing out that he and Andrew McMahon and Cliff Tabin foresaw the potential of the Hedgehog pathway as a drug target in the late 1990s and filed a patent application. Today, the potential of Hedgehog agonists is being energetically explored for directing the differentiation of stem cells, and Hedgehog antagonists are already in clinical use in tumor therapy.

## What's next?

What could possibly be more instructive and thought-provoking than the ideas of prominent research biologists on the most interesting questions still open in their fields? Instructive because you can't phrase the question without providing the context, and thought-provoking - well, obviously.

We asked all the prominent biologists on our Editorial Board for their open questions, and reproduce here the first few that we have published. Profound questions of immune recognition are so lucidly posed by Gillian Griffiths [11] that she will conquer the most immunology-resistant reader; and who wouldn't want to know why a chromosome looks like a chromosome (Frank Uhlmann [12]) and what heterochromatin is for (Susan Gasser [13])? - while Sean Munro [14] lifts the edge of the rug under which we sweep the things we don't want to think about, and asks awkward questions about cells that don't divide, and how our evolution may have been constrained by the need for homeostasis without homeothermy.

I hope that unless by that time Pat Brown and his ilk have succeeded in upending the entire world of scientific publishing, we shall be revisiting these questions in another ten years to see if any have been answered.

## Note

This article is part of the *BMC Biology *tenth anniversary series. Other articles in this series can be found at http://www.biomedcentral.com/bmcbiol/series/tenthanniversary.
